# Microwave-Assisted Synthesis of New *N_1_,N_4_-*Substituted Thiosemicarbazones 

**DOI:** 10.3390/molecules161210668

**Published:** 2011-12-20

**Authors:** Camilla Moretto dos Reis, Danilo Sousa Pereira, Rojane de Oliveira Paiva, Lucimar Ferreira Kneipp, Aurea Echevarria

**Affiliations:** 1 Departamento de Química, Instituto de Ciências Exatas, Universidade Federal Rural do Rio de Janeiro, 23890-000, Seropédica, Rio de Janeiro, Brazil; 2 Departamento de Micologia, Instituto Oswaldo Cruz – FIOCRUZ, 21045-900, Rio de Janeiro, Rio de Janeiro, Brazil

**Keywords:** thiosemicarbazone, thiosemicarbazide, microwave irradiation, antifungal activity

## Abstract

We present an efficient procedure for the synthesis of thirty-six *N*_1_,*N*_4_-substituted thiosemicarbazones, including twenty-five ones that are reported for the first time, using a microwave-assisted methodology for the reaction of thiosemicarbazide intermediates with aldehydes in the presence of glacial acetic acid in ethanol and under solvent free conditions. Overall reaction times (20–40 min when ethanol as solvent, and 3 min under solvent free conditions) were much shorter than with the traditional procedure (480 min); satisfactory yields and high-purity compounds were obtained. The thiosemicarbazide intermediates were obtained from alkyl or aryl isothiocyanates and hydrazine hydrate or phenyl hydrazine by stirring at room temperature for 60 min or by microwave irradiation for 30 min, with lower yields for the latter. The preliminary *in vitro* antifungal activity of thiosemicarbazones was evaluated against *Aspergillus parasiticus *and *Candida albicans*.

## 1. Introduction

Thiosemicarbazones and thiosemicarbazides are now well established as an important class of sulfur/nitrogen donor ligands, particularly for transition metal ions [1,2], because of the remarkably diverse biological activities observed for these compounds. These activities include antiviral [3,4,5], antitumor [6,7], and antimicrobial properties [8], as well as other industrially important activities, including anticorrosion [9] and antifouling [10] effects. Considering all of these properties, it is important to be able to synthesize new series of thiosemicarbazones.

The increasing demand for clean and efficient chemical procedures has been a target for the synthesis of organic compounds. The combined use of microwave irradiation and solvent-free conditions has shown advantages from economic and environmental standpoints [11,12]. Furthermore, microwave-assisted organic reactions generally provide high yields of pure products, minimize the use of organic solvents, and allow for a simplified work-up and shorter reaction times [13].

Our research group has been working on more efficient and cleaner synthetic methods, focusing on microwave irradiation and solvent-free conditions [14,15,16,17]. To extend our investigation and considering the special importance of thiosemicarbazone class, in this paper we report the synthesis of thirty six thiosemicarbazones *N*_1_,*N*_4_-substituted from thiosemicarbazides with low reaction times and good yields by microwave-assisted reactions. Furthermore, the evaluation of antifungal activity against the *Aspergillus parasiticus *and *Candida albicans* was realized.

## 2. Results and Discussion

**Scheme 1:**
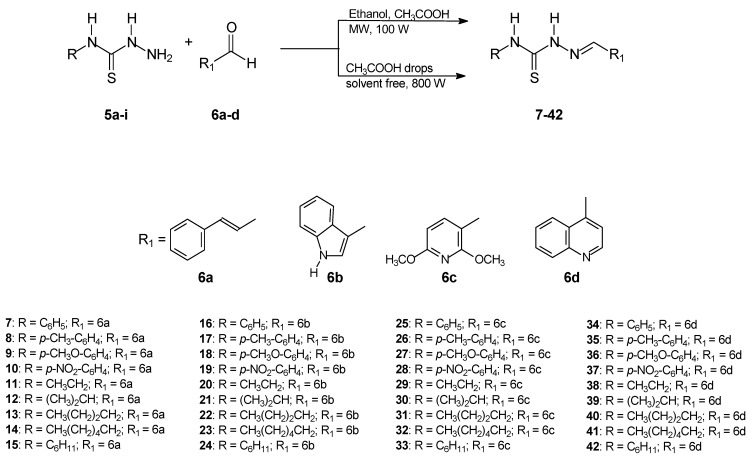
Synthesis of thiosemicarbazones by microwave irradiation.

Thirty-six thiosemicarbazones were synthesized using a microwave-assisted methodology; twenty-five are new compounds. The synthetic procedure was performed in two steps starting with aryl thiosemicarbazides **5a–d** or alkyl thiosemicarbazides **5e–i**, also prepared in this work, and cinnamaldehyde (**6a**), 3-methyl-indolcarboxaldehyde (**6b**), 2,6-dimethoxy-pyridincarboxaldehyde (**6c**) or 4-quinolinecarboxaldehyde (**6d**) in ethanol as a solvent, and under solvent free conditions, with a few drops of added glacial acetic acid, as outlined in Scheme 1. The reaction mixtures were irradiated in a scientific microwave reactor for 20–40 min at 100 W when ethanol used as solvent, and for 3 min at 800 W in the absence of solvent. The products, a cinnamaldehyde series (compounds **7–15**), a 3-methyl-indolcarboxaldehyde series (compounds **16–24**), a 2,6-dimethoxypyridincarboxaldehyde series (compounds **25–33**) and a 4-quinolinecarboxaldehyde series (compounds **34–42**), were obtained with both procedures as fine crystals in high-purity and satisfactory yields after a short time, when compared with the reaction time for the traditional procedure (480 min). The best yields (88–98%) and the short reaction times (3 min) were obtained when the solvent free conditions were used. The thiosemicarbazones **7–10** and **16–19** were also prepared using a reflux method; after 8 h under reflux in ethanol, lower yields were obtained for all the compounds than in the microwave-assisted synthesis. Table 1 shows the reaction times and yields for the target compounds, **7–42**.

**Table 1:** Thiosemicarbazone yields and reaction times obtained under microwave irradiation using ethanol as solvent and in solvent free conditions (**7–42**), and under traditional reflux (**7–10** and **16–19**).

Compound	Time (min)	Yield (%)	Compound	Time (min)	Yield (%)
**7**	3 ^a ^/40 ^b ^/480 ^c ^	96 ^a ^/86 ^b ^/83 ^c ^	**25**	3 ^a ^/40 ^b ^	93 ^a ^/82 ^b ^
**8**	3 ^a ^/40 ^b ^/480 ^c ^	92 ^a ^/79 ^b ^/74 ^c ^	**26**	3 ^a ^/40 ^b ^	86 ^a ^/70 ^b ^
**9**	3 ^a ^/40 ^b ^/480 ^c ^	97 ^a ^/71 ^b ^/70 ^c ^	**27**	3 ^a ^/40 ^b ^	91 ^a ^/78 ^b ^
**10**	3 ^a ^/40 ^b ^/480 ^c ^	90 ^a ^/88 ^b ^/68 ^c ^	**28**	3 ^a ^/40 ^b ^	90 ^a ^/82 ^b ^
**11**	3 ^a ^/20 ^b ^	89 ^a ^/54 ^b ^	**29**	3 ^a ^/20 ^b ^	96 ^a ^/99 ^b ^
**12**	3 ^a ^/20 ^b ^	96 ^a ^/83 ^b ^	**30**	3 ^a ^/20 ^b ^	97 ^a ^/99 ^b ^
**13**	3 ^a ^/20 ^b ^	94 ^a ^/71 ^b ^	**31**	3 ^a ^/20 ^b ^	92 ^a ^/94 ^b ^
**14**	3 ^a ^/20 ^b ^	92 ^a ^/70 ^b ^	**32**	3 ^a ^/20 ^b ^	91 ^a ^/88 ^b ^
**15**	3 ^a ^/20 ^b ^	95 ^a ^/83 ^b ^	**33**	3 ^a ^/20 ^b ^	94 ^a ^/97 ^b ^
**16**	3 ^a ^/40 ^b ^/480 ^c ^	91 ^a ^/62 ^b ^/49 ^c ^	**34**	3 ^a ^/40 ^b ^	89 ^a ^/77 ^b ^
**17**	3 ^a ^/40 ^b ^/480 ^c ^	97 ^a ^/93 ^b ^/78 ^c ^	**35**	3 ^a ^/40 ^b ^	88 ^a ^/84 ^b ^
**18**	3 ^a ^/40 ^b ^/480 ^c ^	88 ^a ^/76 ^b ^/67 ^c ^	**36**	3 ^a ^/40 ^b ^	90 ^a ^/57 ^b ^
**19**	3 ^a ^/40 ^b ^/480 ^c ^	96 ^a ^/82 ^b ^/63 ^c ^	**37**	3 ^a ^/40 ^b ^	96 ^a ^/79 ^b ^
**20**	3 ^a ^/20 ^b ^	98 ^a ^/73 ^b ^	**38**	3 ^a ^/20 ^b ^	95 ^a ^/70 ^b ^
**21**	3 ^a ^/20 ^b ^	93 ^a ^/52 ^b ^	**39**	3 ^a ^/20 ^b ^	93 ^a ^/75 ^b ^
**22**	3 ^a ^/20 ^b ^	94 ^a ^/78 ^b ^	**40**	3 ^a ^/20 ^b ^	91 ^a ^/81 ^b ^
**23**	3 ^a ^/20 ^b ^	87 ^a ^/52 ^b ^	**41**	3 ^a ^/20 ^b ^	89 ^a ^/79 ^b ^
**24**	3 ^a ^/20 ^b ^	95 ^a ^/85 ^b ^	**42**	3 ^a ^/20 ^b ^	93 ^a ^/62 ^b ^

^a^ Solvent free conditions; ^b^ using ethanol as solvent; ^c^ using traditional reflux.

**Figure 1:**
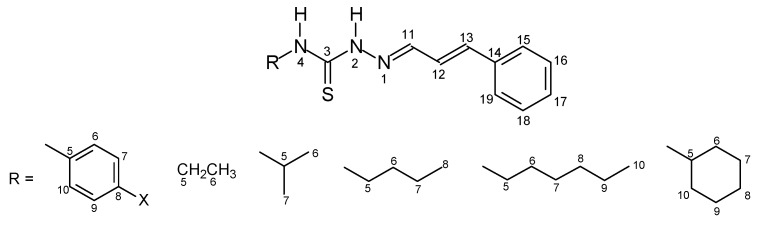
Cinnamaldehyde series **7–15.**

The thiosemicarbazone structures **7–42** were fully characterized by ^1^H- and ^13^C-NMR and IR spectroscopy. The ^1^H- and ^13^C-NMR shifts (δ) were assigned based on literature data [18,19,20,21,22] and were consistent with the structures proposed. The ^1^H chemical shifts of H-C=N for the thiosemicarbazone cinnamaldehyde series products **7–15** showed lower values than for the other series. In contrast, the ^13^C chemical shifts of C=N were assigned with lower and similar values for the 2,6-dimethoxy-3-pyridinecarboxaldehyde and 4-quinolinecarboxaldehyde series products **25–33** and **34–42**. The values ranged from δ = 137.02 to 138.62 for the cinnamaldehyde ones **7–15** and from δ = 140.10 to 146.93 for the 3-indolecarboxaldehyde series **16-24**. The electronic substituent effects were as expected. The Figure 1–Figure 4 show the numbered structures.

**Scheme 2:**
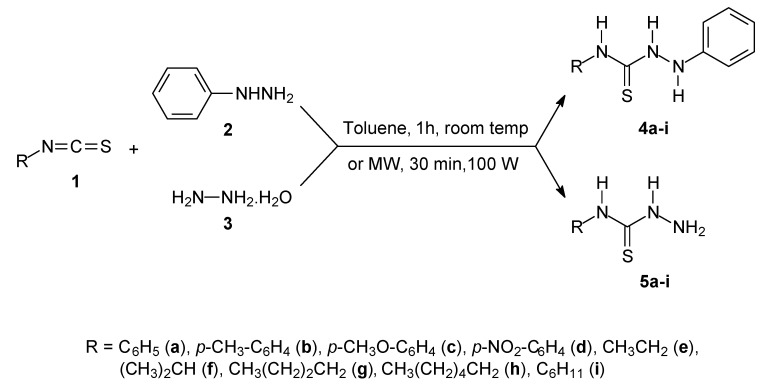
Synthesis of thiosemicarbazides.

The aryl thiosemicarbazides **5a–d** or alkyl thiosemicarbazides **5e–i** used as intermediates in thiosemicarbazone preparation were prepared by both traditional and microwave-assisted procedures. Scheme 2 shows the reactions and the conditions for both procedures.

**Table 2:** Thiosemicarbazide yields and reaction times using microwave irradiation and stirring at room temperature.

Compound	TraditionalProcedure (%) 60 min	Microwave Irradiation (%) 30 min	Compound	TraditionalProcedure (%) 60 min	Microwave Irradiation (%) 30 min
**4a**	93	83	**5a**	79	65
**4b**	92	78	**5b**	85	71
**4c**	92	80	**5c**	80	67
**4d**	95	73	**5d**	89	77
**4e**	97	25	**5e**	97	36
**4f**	99	35	**5f**	99	30
**4g**	94	22	**5g**	94	28
**4h**	94	31	**5h**	94	34
**4i**	93	83	**5i**	98	41

Different from the thiosemicarbazones, thiosemicarbazide preparation via the traditional methodology with toluene as the solvent under stirring at room temperature was more efficient than the microwave-assisted procedure, affording very good yields in comparison, especially for the alkyl isothiosemicarbazides. This performance may be due to the higher volatility of the alkyl isothiocyanates (**1e–i**). However, the reaction times using the microwave irradiation method were 30 minutes, whereas stirring at room temperature required 60 minutes. Table 2 lists the yields in the preparation of **5a–i**. The thiosemicarbazides were identified by comparison with analytical data found in the literature [23].

The preliminary *in vitro* antimicrobial activity of all thiosemicarbazones synthesized on *Aspergillus parasiticus* and *Candida albicans* was evaluated. The thiosemicarbazones showed weak or moderate activity as compared to the standard fungicide itroconazole. However, the observed antimicrobial effects do not exclude from further investigations of these compounds against other fungal strains. Table 3 lists the MIC (μg/mL) values obtained for active thiosemicarbazones.

**Table 3:** MIC (μg/mL) values of thiosemicarbazones against *C. albicans *and *A. parasiticus *isolates.

Compound	*C. albicans*	*A. parasiticus*	Compound	*C. albicans*	*A. parasiticus*
**11**	250	500	**20**	NI	500
**13**	250	500	**21**	250	500
**14**	500	NI	**22**	500	500
**16**	500	500	**23**	NI	500
**17**	NI	500	**24**	500	500
**18**	250	500	**41**	NI	500
**19**	500	500			

NI: no inhibition up to 500 µg/mL.

## 3. Experimental

### 3.1. General

Melting points were determined with a Meltemp II apparatus and were uncorrected. Infrared spectra (KBr pellets) were recorded on a Bruker Vertex 70 spectrophotometer. The ^1^H- and ^13^C-NMR spectra were obtained on a Bruker Avance II 400 spectrometer (^1^H, 400 MHz; ^13^C, 100 MHz) using tetramethylsilane TMS as the internal standard and acetone-*d*_6_ and pyridine-*d*_5_ as the solvent. Elemental analyses were performed on a Perkin-Elmer Model 2400 instrument. The microwave-assisted organic reactions were performed in a CEM Discovery System reactor.

### 3.2. General Procedure for the Preparation of Thiosemicarbazides ***5a–i***

The thiosemicarbazides were prepared according to methods described elsewhere [23]. Briefly, the alkyl or aryl isothiocyanate (25 mmol) and hydrazine hydrate or phenyl hydrazine (25 mmol) were mixed in the presence of toluene (20 mL). The reaction mixture was kept under stirring for 1 hour at room temperature. The solid obtained was filtered and washed with ice-cold toluene. All thiosemicarbazides were identified by the comparison of analytical data (melting points and NMR) with literature reports.

### 3.3. General Procedure for the Preparation of Thiosemicarbazides ***5a–i*** Using Microwave Irradiation

The alkyl or aryl isothiocyanate (0.74 mmol) and hydrazine hydrate or phenyl hydrazine (0.74 mmol) were mixed in the presence of toluene (2 mL) and submitted to microwave irradiation for 30 min at 100 W. The solid obtained was filtered and washed with ice-cold toluene. All thiosemicarbazides were identified by comparing their melting points with those previously obtained.

### 3.4. General Procedure for the Preparation of Thiosemicarbazones ***7–42*** Using Microwave Irradiation and Ethanol as Solvent

The aldehyde (0.84 mmol) and alkyl or aryl thiosemicarbazide (0.84 mmol) were mixed in the presence of ethanol (5 mL) and few drops of glacial acetic acid and submitted to microwave irradiation for 20–40 min at 100 W. The solid obtained was filtered and washed with ice-cold ethanol several times.

### 3.5. General Procedure for the Preparation of Thiosemicarbazones ***7–42*** Using Microwave Irradiation in Solvent-Free Conditions

The aldehyde (0.84 mmols) and alkyl or aryl thiosemicarbazide (0.84 mmols) were mixed in an agate mortar with few drops of glacial acetic acid and submitted to microwave irradiation for 3 min at 800 W. The solid obtained was extracted and recrystallized from ethanol to furnish the pure products.

### 3.6. General Procedure for the Preparation of Thiosemicarbazones ***7–10*** and ***16–19*** Using Traditional Reflux

The aldehyde (1.19 mmol) and aryl thiosemicarbazide (1.19 mmol) were mixed in the presence of ethanol (10 mL) and few drops of glacial acetic acid. The reaction mixture was kept under reflux for 8 hours. The solid obtained was filtered and washed with ice-cold ethanol several times.

*Cinnamaldehyde-4-phenyl-thiosemicarbazone* (**7**)*.* Yellow solid; m.p. 174–176 °C (lit. [18] 176 °C); yield 86%; FT-IR (KBr, υ cm^−1^): 3,271 (N-H), 3,107 (N-H), 1,552 (C=C), 1,517 (C=N), 1,068 (C=S), 991 (C=C); ^1^H-NMR (acetone-*d*_6_) δ 11.80 (s, 1H, H-4), 9.93 (s, 1H, H-2), 7.98 (d, 1H, H-11), 7.60 (d, 2H, H-15 to 19), 7.56 (d, 2H, H-6, H-10), 7.39 (t, 2H, H-7 to 9), 7.30–7.35 (m, 1H, H-17), 7.30–7.35 (m, 2H, H-16, H-18), 7.16 (t, 1H, H-8), 7.06 (d, 1H, H-13), 6.94 (dd, 1H, H-12); ^13^C-NMR (acetone-*d*_6_) δ 177.42 (C-3), 145.81 (C-11), 141.04 (C-17), 140.54 (C-5), 129.62 (C-6, C-10), 128.41 (C-7, C-9), 126.45 (C-8), 126.40 (C-13), 125.24 (C-12), 137.64 (C-14), 130.33 (C-15, C-19), 130.28 (C-16, C-18).

*Cinnamaldehyde-4-(p-methyl-phenyl)-thiosemicarbazone* (**8**)*.* Yellow solid; m.p. 178–180 °C (lit. [19] 176 °C); yield 79%; FT-IR (KBr, υ cm^−^^1^): 3,340 (N-H), 3,118 (N-H), 2,981 (C-H), 1,556 (C=C), 1,517 (C=N), 1,068 (C=S), 981 (C=C); ^1^H-NMR (acetone-*d*_6_) δ 11.85 (s, 1H, H-4), 9.84 (s, 1H, H-2), 7.96 (d, 1H, H-11), 7.55 (d, 2H, H-15, H-19), 7.44 (d, 2H, H-6, H-10), 7.12 (d, 2H, H-7, H-9), 7.32 (t, 1H, H-17), 7.39 (t, 2H, H-16, H-18), 7.05 (d, 1H, H-13), 6.94 (dd, 1H, H-12), 2.28 (s, 3H, CH_3_); ^13^C-NMR (acetone-*d*_6_) δ 175.41 (C-3), 144.77 (C-11), 139.14 (C-17), 136.35 (C-8), 135.90 (C-5), 134.18 (C-14), 128.89 (C-15, C-19), 128.55 (C-16, C-18), 126.91 (C-6, C-10), 125.12 (C-13), 124.74 (C-7, C-9), 20.52 (CH_3_).

*Cinnamaldehyde-4-(p-methoxy-phenyl)-thiosemicarbazone* (**9**). Yellow solid; m.p. 152–154 °C (lit. [19] 155 °C); yield: 71%; FT-IR (KBr, υ cm^−1^): 3,319 (N-H), 3,143 (N-H), 2,985 (C-H), 1,537 (C=C), 1,514 (C=N), 1247 (C-O), 1,201 (O-CH_3_), 1,026 (C=S), 964 (C=C); ^1^H-NMR (acetone-*d*_6_) δ 11.72 (s, 1H, H-4), 9.80 (s, 1H, H-2), 7.96 (d, 1H, H-11), 7.55 (d, 2H, H-15, H-19), 7.41 (d, 2H, H-6, H-10), 6.88 (d, 2H, H-7, H-9), 7.32 (t, 1H, H-17), 7.39 (t, 2H, H-16, H-18), 7.04 (d, 1H, H-13), 6.93 (dd, 1H, H-12), 3.74 (s, 3H, OCH_3_); ^13^C-NMR (acetone-*d*_6_) δ 177.95 (C-3), 158.86 (C-8), 145.60 (C-11), 140.81 (C-17), 137.72 (C-14), 133.45 (C-5), 126.57 (C-12), 127.37 (C-6, C-10), 127.26 (C-13), 130.30 (C-15, C-19), 128.41 (C-16, C-18), 114.82 (C-7, C-9), 56.25 (OCH_3_).

*Cinnamaldehyde-4-(p-nitro-phenyl)-thiosemicarbazone* (**10**). Yellow solid; m.p. 198–199 °C; yield 88%; FT-IR (KBr, υ cm^−1^): 3,263 (N-H), 3,149 (N-H), 1,598 (C=C), 1,562 (C=N), 1,332 (N=O), 1,207 (C=S), 970 (C=C), 850 (C-N); ^1^H-NMR (acetone-*d*_6_) δ 12.12 (s, 1H, H-4), 10.40 (s, 1H, H-2), 8.02 (d, 1H, H-11), 7.58 (d, 2H, H-15, H-19), 8.07 (d, 2H, H-6, H-10), 8.20 (d, 2H, H-7, H-9), 7.34 (t, 1H, H-17), 7.40 (t, 2H, H-16, H-18), 7.11 (d, 1H, H-13), 6.96 (dd, 1H, H-12); ^13^C-NMR (acetone-*d*_6_) δ 176.88 (C-3), 146.93 (C-11), 146.64 (C-8), 145.36 (C-5), 141.98 (C-17), 137.57 (C-14), 130.58 (C-15, C-19), 130.36 (C-16, C-18), 128.53 (C-7, C-9), 126.21 (C-13), 125.35 (C-6, C-10), 124.00 (C-12). Anal. Calcd. for C_16_H_14_N_4_O_2_S (326.37); C, 58.88; H, 4.32; N, 17.17%. Found: C, 58.84; H, 4.39; N, 17.23%.

*Cinnamaldehyde-4-ethyl-thiosemicarbazone* (**11**). White solid; m.p. 166–168 °C; yield 54%; FT-IR (KBr, υ cm^−1^): 3,315 (N-H), 3,134 (N-H), 3,001 (C-H), 1,554 (C=N), 1,523 (C=C), 1,085 (C=S), 977 (C=C); ^1^H-NMR (acetone-*d*_6_) δ 10.28 (s, 1H, H-2), 7.97 (s, 1H, H-4), 7.94 (d, 1H, H-11), 7.55 (d, 2H, H-15, H-19), 7.38 (t, 2H, H-16, H-18), 7.31 (t, 1H, H-17), 6.99 (d, 1H, H-13), 6.88 (dd, 1H, H-12), 3.69 (q, 2H, H-5), 1.21 (t, 3H, H-6); ^13^C-NMR (acetone-*d*_6_) δ 178.57 (C-3), 144.51 (C-11), 140.31 (C-12), 137.05 (C-14), 130.28 (C-16, C-18), 130.19 (C-17), 129.01 (C-15, C-19), 125.31 (C-13), 39.42 (C-5), 14.20 (C-6). Anal. Calcd. for C_12_H_15_N_3_S (233.33); C, 61.77; H, 6.48; N, 18.01%. Found: C, 61.83; H, 6.44; N, 18.11%.

*Cinnamaldehyde-4-isopropyl-thiosemicarbazone* (**12**). White solid; m.p. 195–196 °C; yield 83%; FT-IR (KBr, υ cm^−1^): 3,296 (N-H), 3,126 (N-H), 2,993 (C-H), 1,556 (C=N), 1,546 (C=C), 1,072 (C=S), 974 (C=C); ^1^H-NMR (acetone-*d*_6_) δ 10.25 (s, 1H, H-2), 7.99 (s, 1H, H-4), 7.94 (d, 1H, H-11), 7.55 (d, 2H, H-15, H-19), 7.38 (t, 2H, H-16, H-18), 7.32 (t, 1H, H-17), 6.99 (d, 1H, H-13), 6.88 (dd, 1H, H-12), 6.64 (s, 1H, H-4), 4.56 (m, 1H, H-5), 1.26 (s, 3H, H-7), 1.25 (s, 3H, H-6); ^13^C-NMR (acetone-*d*_6_) δ 177.62 (C-3), 144.59 (C-11), 139.72 (C-12), 137.11 (C-14), 129.67 (C-18), 129.65 (C-16), 129.59 (C-17), 127.75 (C-15, C-19), 125.97 (C-13), 46.58 (C-5), 22.36 (C-6 to 7). Anal. Calcd. for C_13_H_17_N_3_S (247.36); C, 63.12; H, 6.93; N, 16.99%. Found: C, 63.16; H, 6.89; N, 17.04%.

*Cinnamaldehyde-4-butyl-thiosemicarbazone* (**13**). Yellow solid; m.p. 105–107 °C; yield 71%; FT-IR (KBr, υ cm^−1^): 3,278 (N-H), 3,155 (N-H), 2,997 (C-H), 1,552 (C=N), 1,517 (C=C), 1,091 (C=S), 974 (C=C); ^1^H-NMR (acetone-*d*_6_) δ 10.28 (s, 1H, H-2), 7.98 (s, 1H, H-4), 7.94 (d, 1H, H-11), 7.54 (d, 2H, H-15, H-19), 7.38 (t, 2H, H-16, H-18), 7.31 (t, 1H, H-17), 6.99 (d, 1H, H-13), 6.89 (dd, 1H, H-12), 3.65 (q, 2H, H-5), 1.63 (m, 2H, H-6), 1.37 (m, 2H, H-7), 0.93 (t, 3H, H-8); ^13^C-NMR (acetone-*d*_6_) δ 178.76 (C-3), 144.56 (C-11), 139.63 (C-12), 137.73 (C-19), 137.10 (C-14), 129.59 (C-17), 129.26 (C-16, C-18), 127.73 (C-15), 125.97 (C-13), 44.38 (C-5), 30.04 (C-6), 19.76 (C-7), 14.10 (C-8). Anal. Calcd. for C_14_H_19_N_3_S (261.38); C, 64.33; H, 7.33; N, 16.08%. Found: C, 64.28; H, 7.38; N, 7.39%.

*Cinnamaldehyde-4-hexyl-thiosemicarbazone* (**14**). Yellow solid; m.p. 95–97 °C; yield 70%; FT-IR (KBr, υ cm^−1^): 3,346 (N-H), 3,132 (N-H), 2,927 (C-H), 1,560 (C=N), 1,527 (C=C), 1,099 (C=S), 972 (C=C); ^1^H-NMR (acetone-*d*_6_) δ 10.28 (s, 1H, H-2), 7.97 (s, 1H, H-4), 7.94 (d, 1H, H-11), 7.54 (d, 2H, H-15, H-19), 7.38 (t, 2H, H-16, C-18), 7.31 (t, 1H, H-17), 6.99 (d, 1H, H-13), 6.89 (dd, 1H, H-12), 3.64 (q, 2H, H-5), 1.67 (m, 2H, H-6), 1.33 (m, 6H, H-7, H-9), 0.88 (t, 3H, H-10); ^13^C-NMR (acetone-*d*_6_) δ 178.74 (C-3), 144.55 (C-11), 139.62 (C-12), 137.10 (C-14), 129.67 (C-16, C-18), 129.61 (C-17), 127.72 (C-15, C-19), 125.96 (C-13), 44.68 (C-5), 32.26 (C-8), 29.87 (C-6), 27.22 (C-7), 22.20 (C-9), 17.27 (C-10). Anal. Calcd. for C_16_H_23_N_3_S (289.44); C, 66.40; H, 8.01; N, 14.52%. Found: C, 66.47; H, 7.94; N, 14.59%.

*Cinnamaldehyde-4-cyclohexyl-thiosemicarbazone* (**15**). White solid; m.p. 225–226 °C; yield 83%; FT-IR (KBr, υ cm^−1^): 3,275 (N-H), 3,122 (N-H), 2,925 (C-H), 1,546 (C=N), 1,517 (C=C), 1,068 (C=S), 974 (C=C); ^1^H-NMR (pyridine-*d*_5_) δ 12.58 (s, 1H, H-2), 8.38 (s, 1H, H-4), 8.10 (dd, 1H, H-11), 7.47 (d, 2H, H-15, H-19), 7.35 (t, 2H, H-16, H-18), 7.28 (t, 1H, H-17), 6.99 (d, 1H, H-13), 6.86 (dd, 1H, H-12), 4.75 (m, 1H, H-5), 1.64-1.04 (m, 10H, H-6 to H-10); ^13^C-NMR (pyridine-*d*_5_) δ 177.57 (C-3), 143.60 (C-11), 138.87 (C-12), 136.80 (C-14), 129.26 (C-16, C-18), 129.14 (C-17), 127.36 (C-15, C-19), 125.87 (C-13), 55.39 (C-5), 32.94 (C-6, C-10), 25.81 (C-8), 25.44 (C-7, C-9). Anal. Calcd. for C_16_H_21_N_3_S (287.42); C, 66.86; H, 7.36; N, 14.62%. Found: C, 66.81; H, 7.31; N, 14.71%.

**Figure 2:**
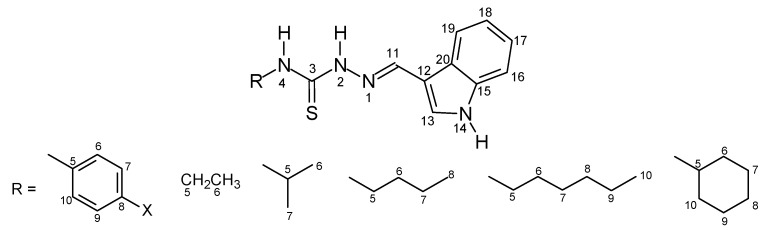
Indole carboxaldehyde series **16–24**.

*Indole-3-carboxaldehyde-4-phenyl-thiosemicarbazone *(**16**). Beige solid; m.p. 201–203 °C (lit. [20] 197–199 °C); yield: 62%; FT-IR (KBr, υ cm^−1^): 3,410 (N-H), 3,317 (N-H), 3,314 (N-H), 1,554 (C=N), 1,105 (C=S); ^1^H-NMR (acetone-*d*_6_) δ 11.60 (s, 2H, H-2, H-14), 9.61 (s, 1H, H-4), 8.40 (s, 1H, H-11), 8.23 (d, 1H, H-19), 7.90 (d, 1H, H-13), 7.63 (d, 2H, H-6, H-10), 7.42 (d, 1H, H-16), 7.37 (t, 2H, H-7, H-9), 7.20 (t, 1H, H-18), 7.14 (t, 1H, H-17); ^13^C-NMR (acetone-*d*_6_) δ 176.82 (C-3), 141.99 (C-11), 140.83 (C-15), 138.95 (C-5), 132.05 (C-13), 129.60 (C-6, C-10), 126.17 (C-8), 125.96 (C-20), 125.24 (C-7, C-9), 124.38 (C-17), 123.08 (C-18), 122.42 (C-19), 113.21 (C-12), 113.25 (C-16).

*Indole-3-carboxaldehyde-4-(p-methyl-phenyl)-thiosemicarbazone* (**17**). Beige solid; m.p. 198–199 °C (lit. [21] 200 °C); yield 93%; FT-IR (KBr, υ cm^−^^1^): 3,404 (N-H), 3,309 (N-H), 3,165 (N-H), 2,920 (C-H), 1,544 (C=N), 1,197 (C=S); ^1^H-NMR (acetone-*d*_6_) δ 11.67 (s, 1H, H-14), 11.54 (s, 1H, H-2), 9.52 (s, 1H, H-4), 8.39 (s, 1H, H-11), 8.20 (d, 1H, H-19), 7.89 (d, 1H, H-13), 7.48 (d, 2H, H-6, H-10), 7.42 (d, 1H, H-16), 7.20 (t, 1H, H-18), 7.15 (d, 2H, H-7, H-9), 7.13 (t, 1H, H-17), 2.30 (s, 3H, CH_3_); ^13^C-NMR (acetone-*d*_6_) δ 176.88 (C-3), 141.81 (C-11), 138.89 (C-15), 138.21 (C-8), 135.71 (C-5), 131.91 (C-13), 130.05 (C-6, C-10), 126.90 (C-20), 126.38 (C-7 to 9), 124.31 (C-17), 123.00 (C-18), 122.33 (C-19), 113.20 (C-16), 113.05 (C-12), 21.38 (CH_3_).

*Indole-3-carboxaldehyde-4-(p-methoxy-phenyl)-thiosemicarbazone* (**18**). Beige solid; m.p. 202–203 °C (lit. [20] 207–209 °C); yield: 76%; FT-IR (KBr, υ cm^−1^): 3,435 (N-H), 3,315 (N-H), 3,203 (N-H), 2,972 (C-H), 1,548 (C=N), 1,245 (C-O), 1,110 (C=S), 1,029 (O-CH_3_); ^1^H-NMR (acetone-*d*_6_) δ 11.67 (s, 1H, H-14), 11.49 (s, 1H, H-2), 9.47 (s, 1H, H-4), 8.39 (s, 1H, H-11), 8.23 (d, 1H, H-19), 7.88 (d, 1H, H-13), 7.44 (d, 2H, H-6, H-10), 7.42 (d, 1H, H-16), 7.19 (t, 1H, H-18), 7.13 (t, 1H, H-17), 6.92 (d, 2H, H-7 to 9), 3.76 (s, 3H, OCH_3_); ^13^C-NMR (acetone-*d*_6_) δ 177.37 (C-3), 158.77 (C-8), 141.78 (C-11), 138.97 (C-15), 133.80 (C-5), 131.90 (C-13), 127.52 (C-6, C-10), 126.00 (C-20), 124.37 (C-17), 123.16 (C-18), 122.37 (C-19), 114.79 (C-7, C-9), 113.24 (C16), 113.22 (C12), 58.25 (OCH_3_).

*Indole-3-carboxaldehyde-4-(p-nitro-phenyl)-thiosemicarbazone* (**19**). Orange solid; m.p. 210–212 °C; Yield: 82%; FT-IR (KBr, υ cm^−1^): 3,367 (N-H), 3,296 (N-H), 3,124 (N-H), 1,550 (C=N), 1,334 (N=O), 1,107 (C=S), 844 (C-N); ^1^H-NMR (acetone-*d*_6_) δ 11.97 (s, 1H, H-14), 11.74 (s, 1H, H-2), 10.08 (s, 1H, H-4), 8.44 (s, 1H, H-11), 8.23 (d, 2H, H-6, H-10), 8.18 (d, 1H, H-19), 8.09 (d, 2H, H-7, H-9), 7.95 (d, 1H, H-13), 7.43 (d, 1H, H-16), 7.21 (t, 1H, H-18), 7.15 (t, 1H, H-17); ^13^C-NMR (acetone-*d*_6_) δ 175.97 (C-3), 146.97 (C-8), 145.16 (C-5), 143.17 (C-11), 139.03 (C-15), 132.67 (C-13), 126.99 (C-20), 125.38 (C-7, C-9), 124.55 (C-17), 123.82 (C-6, C-10), 123.16 (C-18), 122.62 (C-19), 113.38 (C-6), 113.04 (C-12). Anal. Calcd. for C_16_H_13_N_5_O_2_S (339.37); C, 56.63; H, 3.86; N, 20.64%. Found: C, 56.68; H, 3.91; N, 20.69%.

*Indole-3-carboxaldehyde-4-ethyl-thiosemicarbazone* (**20**). Beige solid; m.p. 226–228 °C [22]; yield: 73%; FT-IR (KBr, υ cm^−1^): 3,352 (N-H), 3,238 (N-H), 3,184 (N-H), 2,972 (C-H), 1,546 (C=N), 1,109 (C=S); ^1^H-NMR (pyridine-*d*_5_) δ 12.83 (s, 1H, H-14), 12.29 (s, 1H, H-2), 8.76 (s, 1H, H-11), 8.62 (d, 1H, H-19), 8.51 (s, 1H, H-4),7.89 (d, 1H, H-13), 7.57 (d, 1H, H-16), 7.32 (t, 1H, H-18), 7.23 (t, 1H, H-17), 3.99 (m, 2H, H-5), 1.26 (t, 3H, H-6); ^13^C-NMR (pyridine-*d*_5_) δ 178.10 (C-3), 140.32 (C-11), 138.37 (C-15), 130.85 (C-13), 125.35 (C-20), 123.36 (C-17), 122.44 (C-18), 121.20 (C-19), 112.63 (C-12), 112.45 (C-16), 39.32 (C-5), 15.15 (C-6).

*Indole-3-carboxaldehyde-4-isopropyl-thiosemicarbazone* (**21**). Beige solid; m.p. 156–158 °C (lit. [21] 160 °C); yield: 52%; FT-IR (KBr, υ cm^−1^): 3,346 (N-H), 3,307 (N-H), 3,157 (N-H), 2,922 (C-H), 1,548 (C=N), 1,056 (C=S); ^1^H-NMR (pyridine-*d*_5_) δ 12.86 (s, 1H, H-14), 12.34 (s, 1H, H-2), 8.75 (s, 1H, H-11), 8.55 (d, 1H, H-19), 7.95 (s, 1H, H-4), 7.91 (d, 1H, H-13), 7.59 (d, 1H, H-16), 7.39 (t, 1H, H-18), 7.35 (t, 1H, H-17), 5.00 (m, 1H, H-5), 1.33 (s, 3H, H-6), 1.32 (s, 3H, H-7); ^13^C-NMR (pyridine-*d*_5_) δ 177.21 (C-3), 140.19 (C-11), 130.89 (C-13), 138.46 (C-15), 125.46 (C-20), 123.47 (C-17), 122.03 (C-18), 121.45 (C-19), 112.68 (C-16), 112.62 (C-12), 42.35 (C-5), 22.70 (C-6, C-7).

*Indole-3-carboxaldehyde-4-butyl-thiosemicarbazone* (**22**). Beige solid; m.p. 172–178 °C (lit. [21] 170 °C); yield: 78%; FT-IR (KBr, υ cm^−1^): 3,410 (N-H), 3,365 (N-H), 3,138 (N-H), 2,954 (C-H), 1,537 (C=N), 1,105 (C=S); ^1^H-NMR (pyridine-*d*_5_) δ 12.84 (s, 1H, H-14), 12.34 (s, 1H, H-2), 8.77 (s, 1H, H-11), 8.64 (d, 1H, H-19), 8.45 (s, 1H, H-4), 7.90 (d, 1H, H-13), 7.34 (t, 1H, H-18), 7.58 (d, 1H, H-16), 7.28 (t, 1H, H-17), 3.97 (q, 2H, H-5), 1.71 (m, 2H, H-7), 1.36 (m, 2H, H-6), 0.84 (t, 3H, H-8); ^13^C-NMR (pyridine-*d*_5_) δ 178.31 (C-3), 140.29 (C-11), 130.94 (C-13), 138.45 (C-15), 125.41 (C-20), 123.44 (C-17), 122.45 (C-18), 121.29 (C-19), 112.71 (C-16), 112.56 (C-12), 44.26 (C-5), 32.05 (C-6), 20.43 (C-7), 14.01 (C-8).

*Indole-3-carboxaldehyde-4-hexyl-thiosemicarbazone* (**23**). Beige solid; m.p. 170–172 °C [22]; yield: 52%; FT-IR (KBr, υ cm^−1^): 3354 (N-H), 3,259 (N-H), 3,195 (N-H), 2,925 (C-H), 1,544 (C=N), 1,105 (C=S); ^1^H-NMR (pyridine-*d*_5_) δ 12.84 (s, 1H, H-14), 12.34 (s, 1H, H-2), 8.77 (s, 1H, H-11), 8.65 (d, 1H, H-19), 8.47 (s, 1H, H-4), 7.90 (d, 1H, H-13), 7.58 (d, 1H, H-16), 7.34 (t, 1H, H-18), 7.29 (t, 1H, H-17), 3.99 (q, 2H, H-5), 1.75 (m, 2H, H-6), 1.35 (m, 2H, H-7), 1.21 (m, 4H, H-8, H-9), 0.8 (t, 3H, H-10); ^13^C-NMR (pyridine-*d*_5_) δ 178.25 (C-3), 140.30 (C-11), 138.42 (C-15), 130.96 (C-13), 125.36 (C-20), 123.42 (C-17), 122.43 (C-19), 121.25 (C-18), 112.68 (C-12), 112.53 (C-16), 44.54 (C-5), 31.75 (C-8), 29.60 (C-6), 26.95 (C-7), 22.85 (C-9), 14.06 (C-10).

*Indole-3-carboxaldehyde-4-cyclohexyl-thiosemicarbazone* (**24**). Beige solid; m.p. 203–205 °C; yield: 84%; FT-IR (KBr, υ cm^−1^): 3,410 (N-H), 3,354 (N-H), 3,219 (N-H), 2,923 (C-H), 1544 (C=N), 1,105 (C=S); ^1^H-NMR (pyridine-*d*_5_) δ 12.88 (s, 1H, H-14), 12.39 (s, 1H, H-2), 8.75 (s, 1H, H-11), 8.60 (d, 1H, H-19), 8.03 (s, 1H, H-4), 7.92 (d, 1H, H-13), 7.60 (d, 1H, H-16), 7.44 (t, 1H, H-18), 7.37 (t, 1H, H-17), 4.77 (m, 1H, H-5), 1.65-1.10 (m, 10H, H-6 to H-10); ^13^C-NMR (pyridine-*d*_5_) δ 177.04 (C-3), 140.10 (C-11), 130.98 (C-13), 138.47 (C-15), 123.47 (C-17), 125.41 (C-20), 122.04 (C-18), 121.44 (C-19), 112.69 (C-16), 112.63 (C-12), 52.58 (C-5), 32.96 (C-6, C-10), 25.74 (C-7), 25.02 (C-7, C-9). Anal. Calcd. for C_16_H_20_N_4_S (300.42); C, 63.97; H, 6.71; N, 18.65%. Found: C, 63.94; H, 6.68; N, 18.71%.

**Figure 3:**
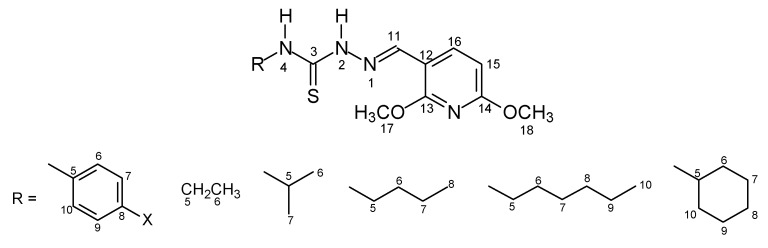
2,6-Dimethoxypyridinecarboxaldehyde series **25–33**.

*2,6-Dimethoxypyridine-3-carboxaldehyde-4-phenylthiosemicarbazone *(**25**). Yellow solid; m.p. 211–212 °C; yield: 82%; FT-IR (KBr, υ cm^−1^): 3,307 (N-H), 3,128 (N-H), 2,978 (C-H), 1,595 (C=N), 1,326 (C-O), 1,205 (O-CH_3_), 1,004 (C=S); ^1^H-NMR (acetone-*d*_6_) δ 11.77 (s, 1H, H-4), 10.06 (s, 1H, H-2), 8.58 (d, 1-H, H-16), 8.32 (s, 1H, H-11), 7.53 (d, 2H, H-6, H-10), 7.35 (t, 2H, H-7, H-9), 7.19 (t, 1H, H-8), 6.45 (d, 1H, H-15), 3.95 (s, 3H, H-17), 3.90 (s, 3H, H-18); ^13^C-NMR (acetone-*d*_6_) δ 175.52 (C-3), 163.64 (C-13), 160.53 (C-14), 139.08 (C-5), 138.62 (C-16), 137.31 (C-11), 127.95 (C-7, C-9), 125.70 (C-6, C-10), 125.14 (C-8), 108.34 (C-12), 102.39 (C-15), 53.52 (C-17), 53.47 (C-18). Anal. Calcd. for C_15_H_16_N_4_O_2_S (316.38); C, 56.95; H, 5.10; N, 17.71%. Found: C, 56.98; H, 5.06; N, 17.79%.

*2,6-Dimethoxypyridine-3-carboxaldehyde-4-(p-methylphenyl)-thiosemicarbazone *(**26**). Yellow solid; m.p. 187–188 °C; yield: 70%; FT-IR (KBr, υ cm^−1^): 3,286 (N-H), 3,140 (N-H), 2,983 (C-H), 1,598 (C=N), 1,126 (C=S); ^1^H-NMR (acetone-*d*_6_) δ 11.72 (s, 1H, H-4), 9.99 (s, 1H, H-2), 8.58 (d, 1H, H-16), 8.31 (s, 1H, H-11), 7.38 (d, 2H, H-6, H-10), 7.14 (d, 2H, H-7, H-9), 6.45 (d, 1H, H-15), 3.94 (s, 3H, H-17), 3.90 (s, 3H, H-18), 2.49 (s, 3H, CH_3_); ^13^C-NMR (acetone-*d*_6_) δ 175.60 (C-3), 163.61 (C-13), 160.50 (C-14), 138.61 (C-16), 137.15 (C-11), 136.53 (C-8), 134.32 (C-5), 128.45 (C-6), 128.43 (C-10), 125.68 (C-7, C-9), 108.38 (C-12), 102.39 (C-15), 53.52 (C-17), 53.46 (C-18), 20.53 (CH_3_). Anal. Calcd. for C_16_H_18_N_4_O_2_S (330.40); C, 58.16; H, 5.49; N, 16.96%. Found: C, 58.21; H, 5.44; N, 17.02%.

*2,6-Dimethoxypyridine-3-carboxaldehyde-4-(p-methoxyphenyl)-thiosemicarbazone *(**27**). Yellow solid; m.p. 189–190 °C; yield: 78%; FT-IR (KBr, υ cm^−1^): 3,329 (N-H), 3,134 (N-H), 2,981 (C-H), 1,604 (C=N), 1,326 (C-O), 1,213 (O-CH_3_), 1,114 (C=S); ^1^H-NMR (acetone-*d*_6_) δ 11.69 (s, 1H, H-4), 9.96 (s, 1H, H-2), 8.58 (d, 1H, H-16), 8.30 (s, 1H, H-11), 7.35 (d, 2H, H-6, H-10), 6.90 (t, 2H, H-7, H-9), 6.44 (d, 1H, H-15), 3.94 (s, 3H, H-17), 3.90 (s, 3H, H-18), 3.75 (s, 3H, OCH_3_); ^13^C-NMR (acetone-*d*_6_) δ 175.94 (C-3), 163.59 (C-13), 160.48 (C-14), 156.86 (C-8), 138.59 (C-16), 137.02 (C-11), 132.00 (C-5), 127.45 (C-6, C-10), 113.20 (C-7, C-9), 108.43 (C-12), 102.38 (C-15), 55.20 (OCH_3_), 53.53 (C-17), 53.46 (C-18). Anal. Calcd. for C_16_H_18_N_4_O_3_S (346.40); C, 55.48; H, 5.24; N, 16.17%. Found: C, 55.41; H, 5.21; N, 16.23%.

*2,6-Dimethoxypyridine-3-carboxaldehyde-4-(p-nitrophenyl)-thiosemicarbazone *(**28**). Yellow solid; m.p. 209–211 °C; yield: 82%; FT-IR (KBr, υ cm^−1^): 3,257 (N-H), 3,122 (N-H), 2,968 (C-H), 1,598 (C=N), 1,332 (N=O), 1,107 (C=S), 844 (C-N); ^1^H-NMR (acetone-*d*_6_) δ 12.12 (s, 1H, H-4), 10.37 (s, 1H, H-2), 8.56 (d, 1H, H-16), 8.37 (s, 1H, H-11), 8.21 (d, 2H, H-7, H-9), 8.04 (d, 2H, H-6, H-10), 6.49 (d, 1H, H-15), 3.95 (s, 3H, H-17), 3.90 (s, 3H, H-18); ^13^C-NMR (acetone-*d*_6_) δ 174.71 (C-3), 163.92 (C-13), 160.79 (C-14), 145.41 (C-8), 143.32 (C-5), 138.69 (C-16), 138.62 (C-11), 124.17 (C-6, C-10), 123.64 (C-7, C-9), 107.99 (C-12), 102.52 (C-5), 53.58 (C-17), 53.54 (C-18). Anal. Calcd. for C_15_H_15_N_5_O_4_S (361.38); C, 49.86; H, 4.18; N, 19.38%. Found: C, 49.81; H, 4.23; N, 19.42%.

*2,6-Dimethoxypyridine-3-carboxaldehyde-4-ethylthiosemicarbazone *(**29**). White solid; m.p. 218–220 °C; yield: 99%; FT-IR (KBr, υ cm^−1^): 3,276 (N-H), 3,138 (N-H), 2,976 (C-H), 1,604 (C=N), 1,380 (C-O), 1,236 (O-CH_3_), 1,012 (C=S); ^1^H-NMR (pyridine-*d*_5_) δ 12.49 (s, 1H, H-2), 8.99 (s, 1H, H-4), 8.61 (s, 1H, H-11), 7.92 (d, 1H, H-16), 6.24 (d, 1H, H-15), 3.98 (m, 2H, H-5), 3.83 (s, 3H, H-17), 3.80 (s, 3H, H-18), 1.28 (t, 3H, H-6); ^13^C-NMR (pyridine-*d*_5_) δ 178.48 (C-3), 164.21 (C-13), 161.15 (C-14), 137.49 (C-16), 137.17 (C-11), 109.01 (C-12), 102.99 (C-15), 53.55 (C-17), 53.51 (C-18), 39.36 (C-5), 15.06 (C-6). Anal. Calcd. for C_11_H_16_N_4_O_2_S (268.33); C, 49.24; H, 6.01; N, 20.88%. Found: C, 49.28; H, 5.93; N, 20.92%.

*2,6-Dimethoxypyridine-3-carboxaldehyde-4-isopropylthiosemicarbazone *(**30**). White solid; m.p. 216–218 °C; yield: 99%; FT-IR (KBr, υ cm^−1^): 3,315 (N-H), 3,151 (N-H), 2,970 (C-H), 1,597 (C=N), 1,325 (C-O), 1,240 (O-CH_3_), 1,018 (C=S); ^1^H-NMR (pyridine-*d*_5_) δ 12.45 (s, 1H, H-2), 8.63 (s, 1H, H-11), 8.43 (s, 1H, H-4), 7.93 (d, 1H, H-16), 6.24 (d, 1H, H-15), 5.09 (m, 1H, H-5), 3.82 (s, 3H, H-17), 3.79 (s, 3H, H-18), 1.32 (s, 3H, H-6), 1.30 (s, 3H, H-7); ^13^C-NMR (pyridine-*d*_5_) δ 177.49 (C-3), 164.23 (C-13), 161.18 (C-14), 137.57 (C-16), 137.40 (C-11), 108.88 (C-12), 102.98 (C-15), 53.54 (C-17), 53.51 (C-18), 46.48 (C-5), 22.39 (C-6, C-7). Anal. Calcd. for C_12_H_18_N_4_O_2_S (282.36); C, 51.05; H, 6.43; N, 19.84%. Found: C, 51.12; H, 6.40; N, 19.89%.

*2,6-Dimethoxypyridine-3-carboxaldehyde-4-butylthiosemicarbazone *(**31**). White solid; m.p. 204–206 °C; yield: 94%; FT-IR (KBr, υ cm^−1^): 3,334 (N-H), 3,132 (N-H), 2,987 (C-H), 1,604 (C=N), 1,332 (C-O), 1,215 (O-CH_3_), 1,008 (C=S); ^1^H-NMR (acetone-*d*_6_) δ 10.28 (s, 1H, H-2), 8.29 (s, 1H, H-11), 8.21 (s, 1H, H-4), 6.36 (d, 1H, H-15), 3.98 (s, 3H, H-17), 3.93 (s, 3H, H-18), 3.65 (q, 2H, H-5),1.91 (m, 2H, H-7), 1.80 (m, 2H, H-6), 0.92 (t, 3H, H-8); ^13^C-NMR (acetone-*d*_6_) δ 178.84 (C-3), 167.93 (C-13), 160.83 (C-14), 141.43 (C-16), 137.29 (C-11), 109.45 (C-12), 104.42 (C-15), 53.92 (C-16, C-18), 44.39 (C-5), 32.17 (C-6), 20.67 (C-7), 14.14 (C-8). Anal. Calcd. for C_13_H_20_N_4_O_2_S (296.39); C, 52.68; H, 6.80; N, 19.90%. Found: C, 52.72; H, 6.76; N, 19.96%.

*2,6-Dimethoxypyridine-3-carboxaldehyde-4-hexylthiosemicarbazone *(**32**). Yellow solid; m.p. 130–132 °C; yield: 88%; FT-IR (KBr, υ cm^−1^): 3,309 (N-H), 3,126 (N-H), 2,952 (C-H), 1,602 (C=N), 1,328 (C-O), 1,238 (O-CH_3_), 1,012 (C=S); ^1^H-NMR (acetone-*d*_6_) δ 10.28 (s, 1H, H-2), 8.87 (t, 3H, H-10), 8.29 (s, 1H, H-11), 8.25 (s, 1H, H-4), 8.21 (d, 1H, H-16), 6.36 (d, 1H, H-15), 3.97 (s, 3H, H-17), 3.93 (s, 3H, H-18), 3.64 (q, 2H, H-5), 1.65 (m, 2H, H-6), 1.32 (m, 6H, H-7 to H-9); ^13^C-NMR (acetone-*d*_6_) δ 178.79 (C-3), 164.96 (C-14), 161.81 (C-13), 138.37 (C-16), 137.26 (C-11), 109.49 (C-12), 103.42 (C-15), 53.92 (C-17, C-18), 44.68 (C-5), 32.39 (C-8), 32.22 (C-9), 29.97 (C-6), 27.24 (C-7), 14.27 (C-10). Anal. Calcd. for C_15_H_24_N_4_O_2_S (324.44); C, 55.53; H, 7.46; N, 17.27%. Found: C, 55.57; H, 7.42; N, 17.34%.

*2,6-Dimethoxypyridine-3-carboxaldehyde-4-cyclohexylthiosemicarbazone *(**33**). Yellow solid; m.p. 241–243 °C; yield: 97%; FT-IR (KBr, υ cm^−1^): 3,332 (N-H), 3,113 (N-H), 2,978 (C-H), 1,602 (C=N), 1,328 (C-O), 1,222 (O-CH_3_), 1,014 (C=S); ^1^H-NMR (pyridine-*d*_5_) δ 12.49 (s, 1H, H-2), 8.63 (s, 1H, H-11), 8.37 (s, 1H, H-4), 8.01 (d, 1H, H-16), 6.29 (d, 1H, H-15), 4.79 (m, 1H, H-5), 3.83 (s, 3H, H-18), 3.80 (s, 3H, H-17), 1.62–0.99 (m, 10H, H-6 to H-10); ^13^C-NMR (pyridine-*d*_5_) δ 177.40 (C-3), 164.27 (C-13), 161.21 (C-14), 137.41 (C-11), 137.70 (C-16), 108.90 (C-12), 103.04 (C-15), 53.56 (C-17), 53.53 (C-18), 53.49 (C-5), 32.88 (C-6, C-10), 25.76 (C-8), 25.48 (C-7, C-9). Anal. Calcd. for C_15_H_22_N_4_O_2_S (322.43); C, 55.88; H, 6.88; N, 17.38%. Found: C, 55.94; H, 6.82; N, 17.42%.

**Figure 4:**
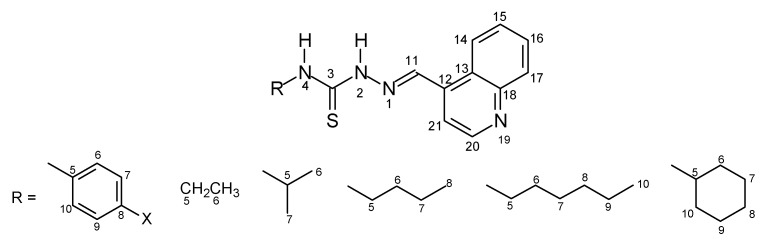
4-Quinolinecarboxaldehyde series **34-42**.

*Quinoline-4-carboxaldehyde-4-phenylthiosemicarbazone *(**34**). Yellow solid; m.p. 195–198 °C (lit. [24] 200 °C); yield: 77%; FT-IR (KBr, υ cm^−1^): 3,338 (N-H), 3,132 (N-H), 1,525 (C=N), 1,195 (C=S); ^1^H-NMR (pyridine-*d*_5_) δ 13.28 (s, 1H, H-2), 11.02 (s, 1H, H-4), 9.09 (s, 1H, H-11), 8.38 (d, 1H, H-14), 8.34 (d, 1H, H-17), 8.06 (d, 2H, H-6, H-10), 7.73 (t, 1H, H-16), 7.72 (d, 1H, H-20), 7.54 (t, 1H, H-15),7.41 (t, 2H, H-7, H-9), 7.22 (t, 1H, H-8), 8.39 (d, 1H, H-19); ^13^C-NMR (pyridine-*d*_5_) δ 178.16 (C-3), 150.39 (C-19), 149.49 (C-18), 140.04 (C-5), 137.79 (C-11), 137.77 (C-12), 130.85 (C-16), 129.74 (C-4), 128.79 (C-9), 128.78 (C-7), 127.52 (C-15), 125.98 (C-6, C-10), 125.95 (C-8), 125.92 (C-13), 123.79 (C-17), 118.53 (C-20).

*Quinoline-4-carboxaldehyde-4-(p-methylphenyl)-thiosemicarbazone *(**35**). Yellow solid; m.p. 203–205 °C; yield: 84%; FT-IR (KBr, υ cm^−1^): 3,319 (N-H), 3,211 (N-H), 2,985 (C-H), 1,544 (C=N), 1,188 (C=S); ^1^H-NMR (pyridine-*d*_5_) δ 13.22 (s, 1H, H-2), 11.00 (s, 1H, H-4), 9.09 (s, 1H, H-11), 8.39 (d, 1H, H-19), 8.38 (d, 1H, H-14), 8.34 (d, 1H, H-17), 7.91 (d, 1H, H-6), 7.88 (d, 1H, H-10), 7.73 (t, 1H, H-16), 7.72 (d, 1H, H-20), 7.54 (t, 1H, H-15), 7.03 (d, 2H, H-7, H-9), 2.19 (s, 1H, H-20); ^13^C-NMR (pyridine-*d*_5_) δ 178.68 (C-3), 150.39 (C-19), 149.51 (C-18), 137.86 (C-8), 137.63 (C-11), 137.46 (C-12), 132.96 (C-5), 130.85 (C-16), 129.74 (C-14), 129.37 (C-6, C-10), 127.97 (C-7), 127.50 (C-15), 126.10 (C-9), 125.94 (C-13), 123.79 (C-17), 118.47 (C-20), 20.87 (CH_3_). Anal. Calcd. for C_18_H_16_N_4_S (320.41); C, 67.48; H, 5.03; N, 17.49%. Found: C, 67.44; H, 4.98; N, 17.54%.

*Quinoline-4-carboxaldehyde-4-(p-methoxyphenyl)-thiosemicarbazone *(**36**). Yellow solid; m.p. 186–189 °C; yield: 57%; FT-IR (KBr, υ cm^−1^): 3,309 (N-H), 3,201 (N-H), 2,929 (C-H), 1,550 (C=N), 1,247 (C-O), 1,184 (C=S), 1,035 (O-CH_3_); ^1^H-NMR (pyridine-*d*_5_) δ 13.23 (s, 1H, H-2), 11.02 (s, 1H, H-4), 9.10 (s, 1H, H-11), 8.87 (d, 1H, H-19), 8.39 (d, 4H, H-14, OCH_3_), 8.34 (d, 1H, H-17), 7.88 (d, 2H, H-7, H-9), 7.72 (d, 1H, H-20), 7.71 (t, 1H, H-16), 7.53 (t, 1H, H-15), 7.01 (d, 2H, H-6, H-10); ^13^C-NMR (pyridine-*d*_5_) δ 178.73 (C-3), 158.12 (C-8), 150.43 (C-19), 149.54 (C-18), 137.90 (C-12), 137.60 (C-11), 132.97 (C-5), 130.89 (C-16), 129.79 (C-14), 128.05 (C-6, C-10), 127.57 (C-15), 126.00 (C-13), 123.52 (C-17), 118.28 (C-20), 114.13 (C-7, C-9), 55.37 (OCH_3_). Anal. Calcd. for C_18_H_16_N_4_OS (336.41); C, 64.27; H, 4.79; N, 16.65%. Found: C, 64.32; H, 4.76; N, 16.72%.

*Quinoline-4-carboxaldehyde-4-(p-nitrophenyl)-thiosemicarbazone *(**37**). Yellow solid; m.p. 220–223 °C; yield: 79%; FT-IR (KBr, υ cm^−1^): 3,296 (N-H), 3,082 (N-H), 1,552 (C=N), 1,330 (N=O), 1,193 (C=S), 852 (C-N); ^1^H-NMR (pyridine-*d*_5_) δ 13.65 (s, 1H, H-2), 11.35 (s, 1H, H-4), 9.13 (s, 1H, H-11), 8.87 (d, 1H, H-19), 8.38 (d, 2H, H-7, H-14), 8.34 (d, 2H, H-9, H-17), 8.26 (d, 1H, H-10), 8.06 (d, 1H, H-6), 7.73 (t, 1H, H-16), 7.65 (d, 1H, H-20), 7.58 (t, 1H, H-15); ^13^C-NMR (pyridine-*d*_5_) δ 177.67 (C-3), 150.39 (C-19), 149.51 (C-18), 146.03 (C-8), 138.97 (C-11), 137.46 (C-12), 130.92 (C-16), 129.83 (C-14), 127.64 (C-15), 125.92 (C-13), 124.73 (C-7, C-10), 124.36 (C-6, C-9), 123.80 (C-17), 118.66 (C-20). Anal. Calcd. for C_17_H_13_N_5_O_2_S (351.38); C, 58.11; H, 3.73; N, 19.93%. Found: C, 58.19; H, 3.68; N, 20.01%.

*Quinoline-4-carboxaldehyde-4-ethylthiosemicarbazone *(**38**). Yellow solid; m.p. 175–177 °C; yield: 70%; FT-IR (KBr, υ cm^−1^): 3,373 (N-H), 3,149 (N-H), 2,958 (C-H), 1,533 (C=N), 1,230 (C=S); ^1^H-NMR (pyridine-*d*_5_) δ 12.86 (s, 1H, H-2), 9.31 (s, 1H, H-4), 9.01 (s, 1H, H-11), 8.84 (d, 1H, H-19), 8.35 (d, 1H, H-14), 8.32 (d, 1H, H-17), 7.69 (t, 1H, H-16), 7.66 (d, 1H, H-20), 7.50 (t, 1H, H-15), 4.01 (m, 2H, H-5), 1.32 (t, 3H, H-6); ^13^C-NMR (pyridine-*d*_5_) δ 179.08 (C-3), 150.34 (C-19), 149.50 (C-18), 138.04 (C-12), 136.94 (C-11), 130.82 (C-16), 129.68 (C-14), 127.41 (C-15), 125.91 (C-13), 123.47 (C-7), 118.22 (C-20), 39.62 (C-5), 14.83 (C-6). Anal. Calcd. for C_13_H_14_N_4_S (258.34); C, 60.44; H, 5.46; N, 21.69%. Found: C, 60.47; H, 5.41; N, 21.73%.

*Quinoline-4-carboxaldehyde-4-isopropylthiosemicarbazone *(**39**). White solid; m.p. 193–195 °C; yield: 75%; FT-IR (KBr, υ cm^−1^): 3,303 (N-H), 3,136 (N-H), 2,976 (C-H), 1,541 (C=N), 1,236 (C=S); ^1^H-NMR (pyridine-*d*_5_) δ 12.86 (s, 1H, H-2), 9.28 (s, 1H, H3), 8.99 (s, 1H, H-11), 8.83 (d, 1H, H-19), 8.72 (s, 1H, H-4), 8.32 (d, 2H, H-14, H-17), 7.70 (t, 1H, H-16), 7.63 (d, 1H, H-20), 7.54 (t, 1H, H-15), 5.09 (m, 1H, H-5), 1.37 (d, 3H, H-7), 1.35 (d, 3H, H-6); ^13^C-NMR (pyridine-*d*_5_) δ 178.15 (C-3), 150.37 (C-18), 149.48 (C-19), 137.97 (C-12), 137.04 (C-11), 130.87 (C-16), 129.69 (C-14), 127.44 (C-15), 125.91 (C-13), 123.42 (C-7), 118.28 (C-20), 46.84 (C-5), 22.23 (C-6, C-7). Anal. Calcd. for C_14_H_16_N_4_S (272.37); C, 61.74; H, 5.92; N, 20.57%. Found: C, 61.77; H, 5.87; N, 20.64%.

*Quinoline-4-carboxaldehyde-4-butylthiosemicarbazone *(**40**). Yellow solid; m.p. 104–108 °C; yield: 81%; FT-IR (KBr, υ cm^−1^): 3,379 (N-H), 3,205 (N-H), 2,927 (C-H), 1,529 (C=N), 1,211 (C=S); ^1^H-NMR (pyridine-*d*_5_) δ 12.89 (s, 1H, H-2), 9.26 (s, 1H, H-4), 9.01 (s, 1H, H-11), 8.86 (d, 1H, H-19), 8.38 (d, 1H, H-14), 8.33 (d, 1H, H-17), 7.70 (t, 1H, H-16), 7.69 (d, 1H, H-20), 7.50 (t, 1H, H-15), 4.00 (m, 2H, H-5), 1.79 (m, 2H, H-7), 1.37 (m, 2H, H-6), 0.85 (t, 2H, H-8); ^13^C-NMR (pyridine-*d*_5_) δ 179.24 (C-3), 150.36 (C-19), 149.50 (C-18), 138.05 (C-12), 137.02 (C-11), 130.82 (C-16), 129.69 (C-14), 127.41 (C-15), 125.88 (C-13), 123.55 (C-17), 118.35 (C-20), 44.56 (C-5), 31.75 (C-6), 20.41 (C-7), 14.00 (C-8). Anal. Calcd. for C_15_H_18_N_4_S (286.39); C, 62.91; H, 6.33; N, 19.56%. Found: C, 62.97; H, 6.28; N, 19.61%.

*Quinoline-4-carboxaldehyde-4-hexylthiosemicarbazone *(**41**). Yellow solid; m.p. 158–160 °C; yield: 57%; FT-IR (KBr, υ cm^−1^): 3,271 (N-H), 3,153 (N-H), 2,927 (C-H), 1,543 (C=N), 1,224 (C=S); ^1^H-NMR (pyridine-*d*_5_) δ 12.89 (s, 1H, H-2), 9.27 (s, 1H, H-4), 9.00 (s, 1H, H-11), 8.85 (d, 1H, H-19), 8.38 (d, 1H, H-14), 8.32 (d, 1H, H-17), 7.70 (t, 1H, H-16), 7.69 (d, 1H, H-20), 7.52 (t, 1H, H-15), 4.00 (q, 2H, H-5), 1.82 (m, 2H, H-6), 1.35 (m, 2H, H-7), 1.20 (m, 4H, H-8, H-9), 0.78 (t, 3H, H-10); ^13^C-NMR (pyridine-*d*_5_) δ 179.28 (C-3), 150.41 (C-19), 149.56 (C-18), 137.10 (C-11), 138.08 (C-12), 130.89 (C-16), 129.73 (C-14), 127.46 (C-15), 125.93 (C-13), 123.60 (C-17), 118.42 (C-20), 44.95 (C-5), 31.80 (C-8), 26.98 (C-7), 22.86 (C-9), 29.70 (C-6), 14.18 (C-10). Anal. Calcd. for C_17_H_22_N_4_S (314.45); C, 64.93; H, 7.05; N, 17.82%. Found: C, 65.02; H, 6.98; N, 17.89%.

*Quinoline-4-carboxaldehyde-4-cyclohexylthiosemicarbazone *(**42**). Yellow solid; m.p. 208–210 °C; yield: 62%; FT-IR (KBr, υ cm^−1^): 3,255 (N-H), 3,174 (N-H), 2,925 (C-H), 1,527 (C=N), 1,207 (C=S); ^1^H-NMR (pyridine-*d*_5_) δ 12.86 (s, 1H, H-2), 9.26 (s, 1H, H4), 8.98 (s, 1H, H-11), 8.90 (d, 1H, H-19), 8.62 (s, 1H, H-4), 8.38 (d, 1H, H-14), 8.34 (d, 1H, H-17), 7.70 (d, 1H, H-20), 7.69 (t, 1H, H-16), 7.56 (t, 1H, H-15), 3.85 (m, 1H, H-5), 1.66-1.04 (m, 10H, H-6 to H-10); ^13^C-NMR (pyridine-*d*_5_) δ 178.05 (C-3), 150.43 (C-19), 149.51 (C-18), 137.95 (C-11), 137.21 (C-12), 130.88 (C-16), 129.72 (C-14), 127.47 (C-15), 125.87 (C-13), 123.55 (C-17), 118.54 (C-20), 53.73 (C-5), 32.64 (C-6, C-10), 35.39 (C-9), 25.74 (C-8), 25.39 (C-7). Anal. Calcd. for C_17_H_20_N_4_S (312.43); C, 65.35; H, 4.45; N, 17.93%. Found: C, 65.41; H, 4.38; N, 18.02%.

### 3.7. Antifungal Activity

Determination of antifungal activity was performed as described in the M27-A2 document of Clinical and Laboratory Standards Institute (CLSI, 2002) for the yeast *Candida albicans* (ATCC 24433) and M38-A for the filamentous fungus *Aspergillus parasiticus *CMT 0334 provided by Mycological Collection of *Trichocomaceae* at IOC/FIOCRUZ-RJ, Brazil. Briefly, the broth microdilution method was performed by 96-well microtiter assay plate containing RPMI 1640 medium (Invitrogen, USA) at pH 7.0 buffered with MOPS 0.16 M. The 36 thiosemicarbazones were diluted in DMSO: Tween 20 (1:1 v/v) to obtain final concentrations ranging from 3.90 to 500 μg/mL and maximum concentration of organic solvent at 2.5%. Next, the yeast *C. albicans* and conidia of *A. parasiticus* suspensions were inoculated into the appropriate well at a final concentration of 0.5–2.5 × 10^3^ CFU mL^−1^ and 0.4–5.0 × 10^4^ CFU mL^−1^, respectively. The minimum inhibitory concentration (MIC) of each drug was determined visually after incubation at 35 °C for 48 h. The lowest concentration inhibiting growth of the organism was recorded as the MIC. Itraconazole (ITC, Sigma Chemical Co., St Louis, MO, USA) was used as reference compound. Each experiment was performed in triplicate.

## 4. Conclusions

In summary, four series of thiosemicarbazones derived from cinnamaldehyde, 3-indole-carboxaldehyde, 2,6-dimethoxypyridinecarboxaldehyde and 4-quinolinecarboxaldehyde and the corresponding thiosemicarbazides were prepared using microwave-assisted reactions in the presence of ethanol as solvent and under solvent free conditions, resulting in good yields, high purity and lower reaction times in comparison with the traditional reflux method, especially when solvent free conditions were utilized.
